# Classification of containers with *Aedes aegypti* pupae using a Neural Networks model

**DOI:** 10.1371/journal.pntd.0006592

**Published:** 2018-07-23

**Authors:** Roberto de Andrade Medronho, Volney de Magalhães Câmara, Leonardo Macrini

**Affiliations:** 1 Faculdade de Medicina, Universidade Federal do Rio de Janeiro, Rio de Janeiro, Brasil; 2 Instituto de Estudos em Saúde Coletiva, Universidade Federal do Rio de Janeiro, Rio de Janeiro, Brasil; 3 Instituto Três Rios, Universidade Federal Rural do Rio de Janeiro, Rio de Janeiro, Brasil; Centers for Disease Control and Prevention, Puerto Rico, UNITED STATES

## Abstract

**Introduction:**

This paper discusses the presence of *Aedes aegypti* pupae in different types of containers considering: volume, pH of the container, among other variables.

**Methods:**

A nonlinear method for selection was applied, based on Mutual Information, by placing in order of importance the most appropriate variables for identifying containers with and without *Aedes aegypti* pupae. Such variables were used for input into a Neural Network in layers for classification.

**Results:**

Among the experiments carried out, the best result obtained used the first eight variables selected by order of importance. The percentage of hits for containers which had no *Aedes aegypti* pupae was 73.3%, and 80.9% for those which did have *Aedes aegypti* pupae in the containers. This Neural Network method, a model with the capacity to emulate non-linear data, got better results in comparison with the discriminant power of the Logistic Regression model. Thus, the outcomes of using the Neural Networks method achieved better separability in classifying the containers with pupae and those with no pupae.

**Conclusion:**

This type of analysis will aid in the efforts to design an efficient program to control *Aedes aegypti* that can concentrate principally on containers which present the greatest productivity.

## Author summary

The authors discuss a nonlinear method, based on Mutual Information, for selection of the presence of *Aedes aegypti* pupae in different breeding sites. The authors compare this method with the logistic regression model. In this study, using the Neural Network model, the variables that better discriminate the containers with *Aedes aegypti* pupae from the containers without pupae were size—the larger the container volume the greater the proportion of containers positive for pupae–, the presence of another type of pupa other than *Aedes aegypti* and the location of the container outside the home. The Neural Network method, a model with the capacity to emulate non-linear data, got better results in comparison with the discriminant power of the Logistic Regression model. Thus, the outcomes of the Neural Networks method achieved better separability in classifying the containers with pupae and those with no pupae. This type of analysis will aid in the efforts to design an efficient program to control *Aedes aegypti* that can concentrate principally on containers which present the greatest productivity.

## Introduction

The incidence of dengue has grown rapidly around the world over the last decades. Batth et al [1] estimated that around 390 million (95% CI: 284–528) dengue infections occurred per year, of which 96 million (95% CI: 67–136) are symptomatic (any level of disease severity). Dengue is currently an important public health problem worldwide. Brady et al [2] estimated that 3.9 billion people, in 128 countries, live in areas at risk for dengue viruses’ infection. In Asia, dengue hemorrhagic fever is predominant in children, while in the Americas adults are more often affected [3,4].

The worldwide emergence of the chikungunya and the Zika viruses and the serious consequences for public health has increased the need for more effective *Aedes aegypti* control programs [5,6,7]. Recently, Brazil has had the largest outbreak of sylvatic yellow fever in recent decades [8] and *Aedes aegytpi* can transmit urban yellow fever.

The emergence of epidemics of dengue, chikungunya, yellow fever, and Zika virus disease is an alert for governments, academia, funders and World Health Organization to improve programs and enhance research in *Aedes*-transmitted diseases [9]. *Aedes aegypti* proliferates in various domestic containers which are used to store water and for ornamental plants. There are also various habitats that receive rain water and may be potential breeding sites for the mosquito, such as used tires, drinking containers, clogged gutters and buildings under construction. Some artificial containers produce large numbers of adult mosquitoes, while others are less productive. Consequently, efforts to control highly productive containers should be priority, especially when resources are limited. This strategy requires extensive knowledge on the ecology of vectors. The control of *Aedes aegypti* is achieved mainly through the elimination of breeding sites which are sites favorable for oviposition that also enable the development of the aquatic phases of the vector. Besides that, control programs for the vector have also sought a greater involvement from the community and have encouraged intersectoral activities [10].

Without effective control of the vector in the American continent, the successive epidemics which are taking place will bring on a depletion of susceptible individuals, and the disease will tend to affect more children. As there is no specific vaccine or specific treatment for dengue [11], the only fragile link in the transmission chain of the disease is its vector, and the best efforts should be taken for its effective control.

A great variety of factors influence the spatial and temporal dynamics of the *Aedes aegypti* and, therefore, the patterns of the dengue transmission in humans. Temperature, rain and humidity interfere at all phases of the development of the vector as well as its dispersion in the environment [12,13].

In underdeveloped countries, the unorganized urbanization, the increased population density, the precariousness of the waste collection and provision of water, along with the inefficiency of the programs for fighting the vector favor the wide expansion of *Aedes aegypti* [14,15,16]. In these countries, the absence or intermittent supply of water drives a large part of the population to store water in various tanks to supply their daily needs. These tanks provide a place for the procreation of the vector in urban areas [14,15]. Other breeding sites propitious for the multiplication of mosquitoes are disposable containers (bottles, cans, plastics etc.), most often found around the outside of homes, and very often inappropriately disposed of, due to the lack of regular waste collection in various areas [17,18,19].

In Brazil, fighting the vector during the transmission periods is sought or achieved through the elimination of potential breeding sites and the application of larvicide in water containers, as well as the use of insecticide for the adult forms [10]. The Ministry of Health in Brazil recommends the indiscriminate removal of containers which have the potential for the reproduction of *Aedes aegypti*, no matter their type or size. However, the feasibility of some small sites to produce adult forms of the vector has not actually been well established. This information is essential for designing a more efficient and cost-effective removal program.

Results obtained from research on the pupae are often used as a proxy to produce adult mosquitoes, as the pupae mortality is considered low [18]. The objective of this study is to investigate the production of *Aedes aegypti* pupae according to the type of use, volume and manufacturing material of the different containers which may potentially be breeding sites for the mosquito, applying non-linear method, and to demonstrate the improvement in results obtained in comparing with linear methods. The identification of the most productive breeding place of the mosquito allows guiding more efficient dengue control program, since concentrates the efforts in the places of greater infestation of the mosquito.

## Materials and methods

### Database

The set of data used in this study was collected in 2004 from the municipality of Nova Iguaçu, state of Rio de Janeiro, Brazil, situated at latitude 22°45’33” South and longitude 43°27’04” West, with a total area of 523,888 m^2^. This county had a population of 750,485 inhabitants and a demographic density of 1,413.8 inhabitants/km^2^. The temperature and the average annual precipitation of rain are 21.8° C and 2,105 mm, respectively.

The Secretary of Health Surveillance of the Ministry of Health carried out a Rapid Survey Index (RSI) of *Aedes aegypti* [20] between November 22 and 26 of 2004. *Breteau* indexes were calculated from the results of this RSI for all quarters of the sample. The Breteau index is calculated as the number of positive containers per 100 houses inspected. The six quarters which presented the greatest *Breteau* indexes were selected for further monitoring. These quarters were in the following neighborhoods: Centro, Califórnia, Vila Operária, Cerâmica, Nova América and Moquetá. All potential breeding sites in the survey delimited region were monitored in a summer week between the 22^sd^ and the 29^th^ December 2004, aiming to identify and collect all immature mosquito specimens. Every container or non-hermetically closed site which contained water (in any volume) found around or inside sample homes during the visits was a potential breeding ground.

Samples of specimens from containers with a capacity of less than 10 liters were collected by aspiration using rubber “pears” or with the help of “shrimp nets”. In the containers with a capacity of over 10 liters, specimens were collected from the drainage system, via the flow of water through the shrimp net. In fixed and large containers, the collection of pupae was carried out by the “sweep net” method, proposed by Tun-Lin *et al* [21], modified by Kubota *et al* [22]. The collected specimens were identified with the aid of binocular bacteriologic microscopes.

The database was composed of 5,954 inspected containers for the presence or absence of *Aedes aegypti* pupae, and the following eleven independent variables:

1. related to the characteristics of the quarter where the containers were located:
  - pluviometric index;
  - temperature;
2. related to the characteristics of the containers:
  - container volume;
  - container pH;
  - presence or absence of other pupae species in the container (the other pupae species were *Aedes albopictus*, *Culex quinquefasciatus*, *Culex nigripalpus*, *Culex coronator*, *Psorophora ciliate*, *Psorophora cilipes*, *Ochlerotatus scapularis*, *Ochlerotatus fluviatilis*, *Limatus durhami* and *Anopheles albitarsis*);
  - location of the container (outside or inside);
  - exposure to sunlight of the container (yes or no)—yes considered partial or total exposition to sunlight during daytime and not only the precise moment of the inspection;
  - containers according mobility, permanence and use (*disposable artificial*–e.g. useless, disposable and removable containers, which depend on the rain water for filling; *artificial in use*–e.g. decorative purposes or for water storage containers as barrels; *permanent for supply*–e.g. water tanks, cisterns, wells; *permanent for water drainage*–e.g. rain gutters; *natural*–e.g. *Bromeliaceae*);
  - type of usage (garbage, water supply, ornamentals, water flowage, domestic use, swimming pool and building foundation);
  - material from which the container was manufactured (plastic/acrylic/expanded polystyrene, metal, ceramics/clay, vulcanized rubber, glass, fiberglass, mineral/masonry/cement, fibrocement/asbestos, organic—animal or vegetal origin material, as egg shells, woods, foliage etc.);
  - size according to the container volume (very small—volume under 250ml, small—volume from 250ml to 1,000ml, medium—volume above 1,000ml to 25,000ml, big—volume above 25,000 to 1,000,000ml, very big—volume above 1,000,000ml).

All existing containers on a 100-meter radius around the center of the six quarters with the highest Breteau indexes were monitored. It was considered as a potential breeding place all non-hermetically closed deposits containing any volume of water. All water holding containers were examined, after oral consent for getting into people’s house. Immature specimens were collected biweekly during monitoring. The breeding sites were also daily monitored between the collections, to verify the presence of pupae, indicating the adults hatching, which would anticipate the interval between the collections (less than fifteen days), to avoid the proliferation of the vector. The problem of an eventual closed house was minimized by the repeated visits.

In this study, the decision to analyze the *Aedes aegypti* pupae is based in the fact that the *Aedes aegypti* larvae mortality is high; on the other hand, when it reaches the pupal stage, it evolves into the adult form giving a better idea about the breeding site productivity.

As previously cited, the objective of this study was to estimate the presence of *Aedes aegypti* pupae considering all the eleven variables cited above in their various containers. The discrimination potential of the selected parameters is assessed in some studies with linear statistical methods, such as the Linear Discriminant Analysis or Principal Component Analysis [23]. In this study, a non-linear method was applied, based on Mutual Information (MI) [24], to select by order of importance the most appropriate variables for distinguishing containers with and without *Aedes aegypti* pupae. These variables were then used as inputs for a Neural Network in layers for classification. For comparison with the proposed model, a logistic regression model was used under the same conditions.

### Data analysis

#### The information theory

Data was analyzed following the principles of the information theory, developed by Shannon in the 1940s for applications in communication engineering. The innovative character of this theory, in alliance with its mathematical elegance, meant it had a great impact not only on engineering but also on various areas, such as statistics and economy.

This section reunites theoretical foundations of the information theory, which are expressed in a descriptive manner. For demonstrations and explanations about the subject, consulting Cover & Thomas [24] is recommended.

#### Shannon’s entropy and mutual information [24]

Uncertainty characterizes the gain in information that the occurrence of an event may provoke. It may, however, be translated through the probability of occurrence of the event. When the occurrence of an event is certain this does not add anything to the information, as all the information is already contained in the certainty of its occurrence. In this way, it can be said that the determination of the quantity of information produced by the occurrence of an event is determined by the quantity of “surprise” that this occurrence brings.

Entropy in the information theory corresponds, however, to the probabilistic uncertainty associated to a distribution of probability.

Definition 1 –Shannon’s entropy *H(X)* of a discrete random variable *X*, with the mass probability function *f*_*x*_*(x)*, is defined by:
  $$H(X)=-\sum_{x\in X}f_{x}(x)\text{log}f_{x}(x)$$ (1)
  where log is to the base 2 and entropy is expressed in bits.
  Thus, by definition, *H(X)* > 0.
Definition 2 –The mutual information (Shannon’s) *I(X;Y)* between two discrete random variables *X* and *Y*, with joint mass probability function *f*_*xy*_*(x*,*y)* and marginal mass probability functions *f*_*x*_*(x)* and *f*_*y*_*(y)* being given by the relative entropy between the joint distribution and the distribution of the marginal product is defined by:
  $$I(X,Y)=\sum_{x\in X}\sum_{y\in Y}f_{xy}(x,y)\text{log}\frac{f_{xy}(x,y)}{f_{x}(x)f_{y}(y)}$$ (2)
  where log is to the base 2 and entropy is expressed in bits.
  with equality if and only if, *f*_*xy*_*(x*,*y)* = *f*_*x*_*(x) f*_*y*_*(y)* (that is, if *X* and *Y* are independent).

From Eq (2), it can be said that mutual information is a measure of statistical independence. The greater the mutual information, the more related the variables are.

#### Selection of variables based on the mutual information feature selector under Uniform Distribution (MIFS-U)

The selection of input variables plays an important role in classification systems such as artificial neural networks (ANNs). Such variables can be classified as pertinent, irrelevant or redundant, and, from the point of view of management of a set of data, which may be gigantic, reducing the number of variables, selecting only those pertinent ones, is extremely desirable. This way, better performance with less computational effort is expected [25].

In 1989 Hosmer & Lemeshow [26] highlighted the importance of the selection of variables, indicating that with a lower number of variables, the model tends to be more generalizable and robust.

The algorithm used in this study is the MIFS-U—Mutual Information Feature Selector under Uniform Distribution—presented in 2002 by Kwak & Choi [25]. It is aimed at overcoming the limitation of the variable selector proposed in 1994 by Battiti [27], generating improved performance in the process of selection. Because of its simplicity, such an algorithm can be used in any classification system, no matter what the learning algorithm is. Input variables can be classified as relevant, irrelevant, or redundant, and you just want to select those that are relevant. The algorithm is initialized with a set F that contains all the variables to be selected and a set S, initially empty, that will be filled for each variable selected in order of importance with the outcome (presence or absence of pupae). The first variable to be selected will be the one that presents the most mutual information with the outcome. Selecting the next variable occurs by choosing a variable *ϕ*_*i*_ ∈ *F* that maximizes $I(C,\phi_{i})-\beta \sum_{\phi_{s}\in S}\left(I(C;\phi_{s})/H(\phi_{s}))I(\phi_{i};\phi_{s}\right)$ and making F←F-{*ϕ*_i_}, S ← *ϕ*_i_. This process repeats itself until F is an empty set. If β = 0, the algorithm selects variables in the order of mutual information between input and output variables. Redundancy among input variables is never reflected. When β>0, the algorithm deletes redundant variables more effectively. In general, we can fix β = 1 [28]. For all the experiments of this study was set β = 1.

#### Neural Network

The variables selected by the MIFS-U algorithm were used as input for a Feedforward Neural Network (FNN). The type of learning used was supervised, that is, using a set of pairs input and output—previously known and that represent the reality. According to Haykin [29], "learning" (or "training") is the process by which the free parameters of a Neural Network are adapted, through a mechanism of presentation of stimuli provided by the environment in which the Network is inserted. The type of training is defined by the way in which the parameters are modified.

The Regularization Bayesian method [30] was used with the intention of reducing the arbitrariness of the specification of the neural architecture. In this method, an objective function is optimized forcing the pruning of less relevant weights. The function *trainbr* of MATLAB is a network training function that updates the weight and bias values according to Levenberg-Marquardt optimization. It minimizes a combination of squared errors and weights, and then determines the correct combination so as to produce a network that generalizes well. Among all the simulations performed, the number of effective neurons used by this training algorithm was more than sufficient [30, 31]. A hidden layer with 10 neurons and an output layer with 1 neuron were used. In all the neurons the function of logistic activation was used. In the output layer the network produced zero or one output, representing the two classes of pupae in the containers, absence or presence of pupae. The data set was divided into training data set (70%) and generalization dataset (30%). The data used in the training were balanced to avoid any tendency favorable to the greater number of cases, that is, absences or presence of pupae. All results presented in this article are related to the generalization dataset. That is, data that were not presented in the training.

#### Logistic regression

Logistic regression has become a standard technique, above all in the medical field, by relating a set of independent variables to a single binary response variable. This is a widely disseminated technique [32].

In many studies the qualitative variable response has two possibilities and, therefore, may be represented by the indicator variable, receiving the values 0 (zero) and 1 (one).

The basic idea behind a logistic regression model consists of minimizing the number of variables so that the resulting model is numerically more stable and easily generalized, given that the more variables included in the model, the more it becomes dependent on the data. Therefore, in many situations, it becomes necessary to use the stepwise techniques in the logistic regression, the process by which variables are included or excluded from the model, based only on statistical criteria such as G statistics (<0,05) and the Wald test (<0,05).

The performance of the models was evaluated through sensitivity (the proportion of true positives that is correctly identified by the model) and specificity (the proportion of true negatives that are correctly identified by the model).

## Results

Table 1 presents the Breteau indexes of the quarters studied. It is noted that the Cerâmica quarter, located in slum, has the highest Breateau index (283.3), while the Vila Operária quarter, located in an urbanized area with a supply of water and garbage collection, presented the lowest Breteau index (90.8).

**Table 1:** *Breateau* index (BI) in the quarters investigated, Nova Iguaçu, Brazil.

Quarter	BI
Centro	125
Califórnia	137.5
Vila Operária	90.8
Cerâmica	283.3
Nova América	133
Moquetá	137.5

Table 2 presents the results of the selection and ordering of variables in relation to the outcome (presence or absence of *Aedes aegypti* pupae), as obtained by the MIFS-U algorithm.

**Table 2:** Results of the selection and ordering of variables for the presence or absence of *Aedes aegypti* pupae according the container categories, Nova Iguaçu, Brazil.

Order of Importance	Container Categories
1	Size
2	Presence of other pupae
3	Location of the container
4	Exposure to sunlight
5	Manufacturing material
6	Mobility and permanence
7	Type of usage
8	Quarter pluviometric index*
9	Container volume
10	Container pH
11	Quarter temperature*

*This category refers to the quarter where the containers were located.

The first three most relevant variables in order of importance for the presence or absence of pupae took size into consideration (categorization of container volume), presence or absence of another type of pupae apart from *Aedes aegypti*, and the location of the container (outside or inside). On the other hand, the less important variables in relation to the presence or absence of *Aedes aegypti* pupae were the container pH and the quarter temperature.

Table 3 presents the performance of the proposed method (Neural Networks) regarding the number of variables used. The data base is made up of information on 5,954 containers, where 5,560 had no pupae and 394 had *Aedes aegypti* pupae. Of these 5,954 containers, 70% were used for training the network and the other 30% were used for testing; that is, the network was trained with 3,857 containers with no pupae and 279 containers with pupae, in a total of 4,136 containers. The containers used for testing (apart from the training sample) were made up of 1,703 with no pupae and 115 with pupae. The following results presented refer to the containers not included in the training sample.

**Table 3:** Experiments related to the optimization of the number of variables and percentage of hits of the presence or absence of pupae in the containers, Nova Iguaçu, Brazil.

	Experiment							
	1	2	3	4	5	6	7	8
Hits	1 to 3	1 to 4	1 to 5	1 to 6	1 to 7	1 to 8	1 to 9	1 to 10
**Specificity**	63.4	70.5	70.8	70.1	68.3	**73.3**	72.4	77.5
**Sensitivity**	67.0	62.6	68.7	74.8	78.3	**80.9**	74.8	71.3
**Accuracy**	63.6	70.0	70.6	70.4	68.9	**73.8**	72.6	77.1

Eight experiments were carried out comparing the first variable on Table 2 with a varying number of the other variables taking into consideration the order of importance. Comparing the results of these 8 experiments it can be observed, for example, that an increase in the ‘container volume’ (ninth variable in order of importance) effectively introduces a worse result regarding predictability of presence of *Aedes aegypti* pupae. The best prediction of the presence of *Aedes aegypti* pupae is obtained in experiment 6, comparing variable 1 with the following 8 variables selected by the MIFS-U algorithm. In this experiment percentage of hits in containers that had no *Aedes aegypti* pupae was 73.3% (specificity) and 80.9% in containers with *Aedes aegypti* pupae (sensitivity), with an accuracy of 73.8%. For this experiment, considering the prevalence of containers with *Aedes aegypti* pupae of 6.3%, the negative predictive value of the Neural Network was 98.3% and the positive predictive value was 17,0%.

A logistic regression was also carried out in the same way, for the purposes of comparison, the results of which are shown in Table 4. A percentage of 63.6% of hits was found in the containers that did not have any *Aedes aegypti* pupae (specificity) and 71.7% in the containers with *Aedes aegypti* pupae (sensitivity), with accuracy of 64.1%. Thus, the capacity for predicting the presence of *Aedes aegypti* pupae of this method was found to be inferior to the method proposed in this study. Considering the prevalence of containers with *Aedes aegypti* pupae of 6.3%, the negative predictive value of the logistic regression was 97.1% and the positive predictive value was 11.7%.

**Table 4:** Results of the analysis considering the logistic regression in the presence or absence of *Aedes aegypti* pupae, Nova Iguaçu, Brazil.

Hits (%)	1 to 8
Specificity	63.6
Sensitivity	71.7
Accuracy	64.1

For coherence, the logistic regression analysis concentrated on the same set of data considered in the same 8 variables selected where the result from the Artificial Neural Network was more significant, that is, size of the container, presence of other pupae in the container, location of the container, the container’s exposure to sunlight, manufacturing material of the container, mobility and permanence, type of usage and quarter pluviometric index.

## Discussion

Using the Neural Network model, the variables that better discriminate the containers with *Aedes aegypti* pupae from the containers without pupae were size—the larger the container volume the greater the proportion of containers positive for pupae–, the presence of another type of pupa other than *Aedes aegypti* and the location of the container outside the home. Arunachalam et al [33] found that the most productive breeding sites for *Aedes aegypti* were the containers of water located outdoors, principally those that were uncovered, under trees and not having been used for at least a week. Areas around and inside the homes were much more important to produce pupae than commercial and public areas. While Martins et al [34] found no significant association between volume of the breeding place and infestation by *Aedes aegypti*, they did find however that the absence of immature forms of *Aedes albopictus* and *Culex spp* in the breeding sites favors its infestation by *Aedes aegypti*.

The Neural Network method, a model with the capacity to emulate non-linear data, got better results in comparison with the discriminant power of the Logistic Regression model. Thus, the outcomes of the Neural Networks method achieved better separability in classifying the containers with pupae and those with no pupae.

According to Medronho *et al* [35], the containers with a greater percentage of pupae are tires, barrels, cisterns, drums and water tanks. In that study, most containers cited (with the exception of tires) are all concerned with the problem of the region’s supply of water. Romero-Vivas *et al* [36] found similar results for the containers used for storage of the supply of water, whereas Barrera *et al* [37] found a larger proportion of containers related to waste positive for pupae.

The containers with the largest proportion of immature forms of the vector were denominated by Tun-Lin *et al* [38] as key containers and according to these authors they should be prioritized in the activities of vector control to make the program more efficient. In this sense, the efforts of an efficient program for controlling *Aedes aegypti* should be primarily concentrated on being able to identify the containers which present greater productivity. We have shown here that the use of a Neural Networks model to aid in monitoring various types of containers can significantly help achieve this goal.

Literature shows that Neural Network have been successfully considered for solving many medical problems, including the prediction of the occurrence of dengue cases [39], the risk classification of dengue patients [40, 41] and the identification and classification of species of the *Anopheles*, *Aedes*, *and Culex*, based on wing shape characters with identification of 100% of *Aedes aegypti* [42]. However, the identification of the most productive breeding sites of *Aedes aegypti* using Neural Network is relatively new and we did not find published articles in this field.

Identification of the most productive breeding sites and their elimination or appropriate treatment may contribute to a more effective mosquito control program.

## Supporting information

S1 Database (XLSX)
